# Overexpression of CXCR2 predicts poor prognosis in patients with colorectal cancer

**DOI:** 10.18632/oncotarget.16086

**Published:** 2017-03-10

**Authors:** Jingkun Zhao, Baochi Ou, Hao Feng, Puxiongzhi Wang, Shuai Yin, Congcong Zhu, Shenjie Wang, Chun Chen, Minhua Zheng, Yaping Zong, Jing Sun, Aiguo Lu

**Affiliations:** ^1^ Shanghai Minimally Invasive Surgery Center, Ruijin Hospital, Shanghai Jiaotong University School of Medicine, Shanghai, PR China; ^2^ Shanghai Institute of Digestive Surgery, Shanghai, PR China; ^3^ Department of General, Visceral, Transplantation, and Vascular Thoracic Surgery, Hospital of University of LMU Munich, Munich, Germany

**Keywords:** colorectal cancer, CXCR2, prognosis, CXCL5

## Abstract

Colorectal cancer is a heterogeneous disease. Although many risk factors are used to predict colorectal cancer patients’ prognosis after surgical resection, new prognostic factors are still needed to be defined to promote predictive efficacy of prognosis and further guide therapies. Herein, we identified the prognostic significance of CXCR2 in colorectal cancer patients. We retrospectively analysed 134 patients with colorectal cancer who underwent minimally invasive surgery between 2010 and 2011. The overall cohort was divided into a training set (*n* = 78) and a validation set (*n* = 56). We detected CXCR2 expression using immunohistochemical staining and defined the cut-off value using X-tile program. Next, we analysed the association between CXCR2 expression and clinicopathologic features in training and validation sets. High expression of CXCR2 was associated with Dukes stage (*P* = 0.018), tumor invasion (*P* = 0.018) and liver metastasis (*P* = 0.047). Multivariate COX regression analyses confirmed that high CXCR2 level was an independent prognostic risk factor for both overall survival and disease free survival. Kaplan-Meier survival analysis demonstrated that patients with high expression of CXCR2 had a poor overall survival and disease free survival even in low-risk group (I + II). This indicated that CXCR2 can help to refine individual risk stratification. In addition, we established Nomograms of all significant factors to predict 3- or 5-years overall survival and disease free survival. Moreover, we found the combination of CXCR2 and its ligand CXCL5 had more significant value in predicting the prognosis than single CXCR2 factor.

## INTRODUCTION

Colorectal cancer (CRC) is one of the most common cancer and leading cause of cancer-related death worldwide, it accounts for approximately 10.0% incidence in male cancer and 9.2% incidence in female cancer [1]. Despite advances in diagnosis and treatment, 5-year survival of CRC patients remains only 60% [2]. As the major cause of death for most cancer patients, tumor metastasis is an important adverse factor in the treatment and prognosis of CRC patients [3]. Local and distant fatal metastasis occurs in 20–25% of these patients after surgery [4]. Traditionally, the risk of metastasis in CRC has been clinically identified by TNM staging, lesions with poor histological differentiation, intestinal obstruction or perforation. However, these clinico- pathological risk factors do not fully distinguish between patients who have a high or low risk of disease reoccurrence. Novel classification systems tends to be based on complex mutational profiles or gene expression patterns of CRC lesions [5]. In addition, for most patients with local or (and) distant disease reoccurrence, the main therapeutic options are systemic chemotherapy, radiotherapy, or both [4]. Since currently used chemotherapy and radiotherapy have poor efficacy in the metastatic stage, for these patients, treatment-resistant disease progression usually leads to tumour-related death within a year of treatment. Therefore, novel molecular biomarkers for the identification of patients at high risk of metastasis and prediction of clinical outcomes as well as molecular-targeted therapeutic approaches are required to further explore.

Chemokines are signaling molecules which bind to G-protein-coupled receptors on target cells. They participate in many biological processes such as organ development, angiogenesis, immune responses as well as tumorigenesis and metastasis [6]. Previously, we elucidated the function of CXCL5 (data are not online) and CCR4 on CRC progression [7]. However, the role of its receptor CXCR2, which expresses in tumor cells and neutrophils membrane was not clarified in our previous study. CXCR2 plays an important role on tumor progression in several types of cancer such as prostate cancer [8], gastric cancer [9] and lung cancer [10]. Additionally, it has been verified that CXCR2-positive neutrophils can be induced by mesenchymal stromal cells to facilitate cancer metastasis in tumor lesions [11].

Here in our study, we investigated the expression of CXCR2 in CRC tissues and analysed the correlation between CXCR2 and clinicopathological characteristics. We found that high- expression of CXCR2 was associated with tumor infiltration and metastasis. CXCR2 acted as a predictor of CRC patients’ survival. Patients with high-expressed CXCR2 tended to have a poor prognosis. Moreover, we constructed Nomogram prediction model and verified the combination of CXCL5 and CXCR2 was also a potent predictor of CRC patients’ prognosis.

## RESULTS

### CXCR2 is high-expressed in CRC tissue and its correlation with clinicopathological parameters

**Figure 1:**
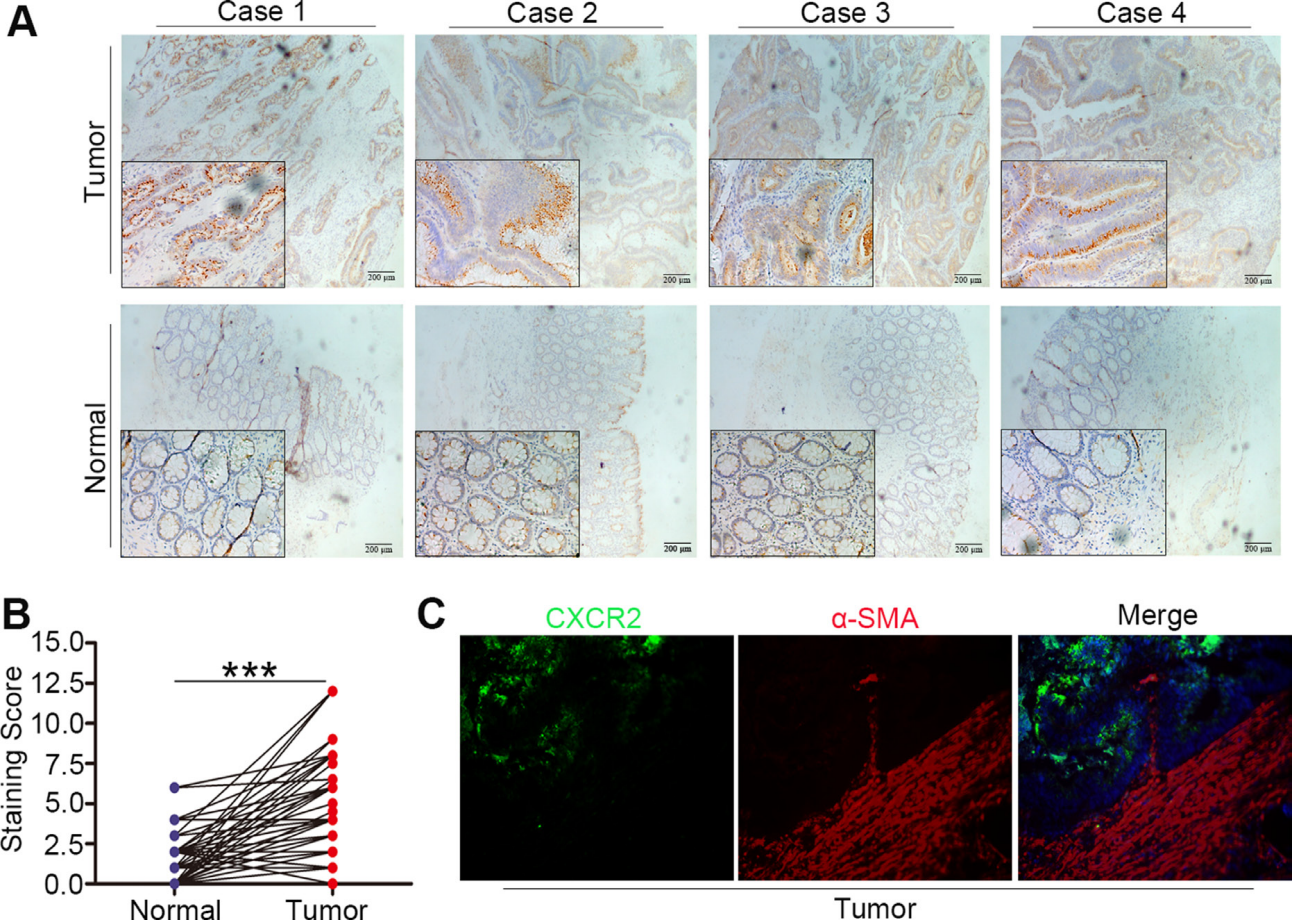
Increased expression and location of CXCR2 in CRC tissue (**A** and **B**) Immunohistochemical results showing high expression of CXCR2 in CRC tissues, the difference between tumor and peritumoral normal tissues is statistically significant (***P < 0.001). (**C**) Immunofluorescence images showing the location of CXCR2 and αSMA in CRC specimens.

Immunohistochemistry assay was performed to investigate the expression of CXCR2 in CRC tissues. The overall cohort of 134 CRC patients was subdivided into a training set (*n* = 78) and a validation set (*n* = 56). As shown in Figure 1A and 1B, CXCR2 was high-expressed in CRC tissues compared with peritumoral normal tissues. In this 134 CRC patients cohort, immunohistochemical results revealed that CXCR2 expression was upregulated in 61.2% (82/134) tissues of overall cohort, 57.7% (45/78) tissues in training set and 66.1% (37/56) tissues in validation set. In addition, CXCR2 was mainly expressed along tumor cell membrane. There was no obvious staining of CXCR2 in tumor mesenchyme (Figure 1A). Immunofluorescence was performed to further verify the location of CXCR2 and tumor mesenchyme was counterstained by α-SMA. Similarly, we found that CXCR2 was mainly expressed in tumor lesions instead of mesenchyme (Figure 1C). Next, we analysed association between CXCR2 expresssion and clinical features of CRC patients. In training set, clinicopathological materials analysis demonstrated that high-expression of CXCR2 in CRC samples was significantly correlated with Dukes stage (*P* = 0.027), tumor invasion (*P* = 0.031) and liver metastasis (*P* = 0.015). Consistently, results from validation set also revealed the correlation between high expression of CXCR2 and Dukes stage (*P* = 0.018), tumor invasion (*P* = 0.018) and liver metastasis (*P* = 0.047). However, high expression of CXCR2 has no correlation with gender, age, histology, tumor location, tumor size and CEA (Table 1).

**Table 1:** Compare between CXCR2 expression and clinicopathologic variables in CRC patients

	Traning Set				Validation Set			
Clinicopathologic Parameters	CXCR2		Statistical Value	*P* Value	CXCR2		Statistical Value	*P* Value
	High	Low			High	Low		
Gender			2.359	0.125			0.214	0.644
Male	27	14			21	12		
Female	18	19			16	7		
Age			0.464	0.496			2.024	0.155
< 65	28	18			19	6		
≥ 65	17	15			18	15		
Histology			1.411	0.494			0.594	0.743
Tubular	37	29			32	16		
Mucinous	7	4			3	1		
Papillary	1	0			2	2		
Tumor location			3.153	0.369			3.118	0.374
Right hemicolon	14	11			10	3		
Transverse colon	1	0			2	1		
Left hemicolon	5	1			6	7		
Sigmoid+Rectum	25	21			19	8		
Tumor size			0.907	0.341			0.714	0.398
≥ 5 cm	24	14			18	7		
< 5 cm	21	19			19	12		
Dukes stage			1.928	0.027			2.088	0.018
I	6	8			1	2		
II	15	12			12	10		
III	18	13			19	6		
IV	6	0			5	1		
TNM stage			1.861	0.031			2.094	0.018
T_1_	4	4			1	2		
T_2_	2	5			8	8		
T_3_	7	9			24	8		
T_4_	32	15			4	1		
N_0_	22	20	1.441	0.075	13	12	1.105	0.135
N_1_	11	9			18	3		
N_2_	12	4			6	4		
M_0_	39	33	2.183	0.015	33	19	1.679	0.047
M_1_	6	0			4	0		
CEA			1.052	0.305			1.504	0.220
≥ 5 ng/mL	23	13			17	12		
< 5 ng/mL	22	20			20	7		

### CXCR2 is an independent prognostic risk factor and Kaplan Meier survival analysis

Next we investigated whether CXCR2 expression was an independent prognostic risk factor in CRC patients. COX regression hazard model was used to perform univariate and multivariate analysis. In traning set, univariate analysis indicated that only Dukes stage and high expression of CXCR2 were risk factors to CRC patients overall survival (OS) and disease free survival (DFS). Multivariate analysis showed that CXCR2 was an independent prognostic factor for both OS and DFS (Table 2). This is consistent with the results of validation set (Table 3).

**Table 2:** Univariate and multivariate analyses of CXCR2 expression of CRC patients in traning set

Parameters	OS			DFS		
	HR	95% CI	*P*	HR	95% CI	*P*
**Univariate analysis**						
Age (< 65vs ≥ 65, year)	1.003	0.410–2.454	0.995	0.794	0.355–1.773	0.573
Gender(female vs male)	1.812	0.740–4.433	0.193	2.190	0.955–5.024	0.064
Histology						
Papillary	1.000	..	0.222	1.000	..	0.231
Tubular vs Papillary	0.164	0.021–1.272	0.084	0.169	0.022–1.305	0.088
Mucinous vs Papillary	0.198	0.020–1.948	0.165	0.167	0.017–1.657	0.126
Location						
Sigmoid+Rectum	1.000	..	0.365	1.000		0.253
Right hemicolon	1.520	0.566–4.084	0.406	1.985	0.826–4.773	0.126
Transverse colon	0.000	0.000–..	0.982	0.000	0.000–..	0.982
Left hemicolon	2.906	0.893–9.449	0.076	2.911	0.908–9.333	0.072
Dukes stage						
IV	1.000	..	**0.002**	1.000	..	**0.001**
I vs IV	0.086	0.016–0.449	**0.004**	0.067	0.013–0.335	**0.001**
II vs IV	0.194	0.064–0.593	**0.004**	0.161	0.056–0.460	**0.001**
III vs IV	0.107	0.028–0.407	**0.001**	0.153	0.050–0.463	**0.001**
Tumor size(< 5 vs ≥ 5, cm)	0.537	0.219–1.314	0.173	0.550	0.244–1.240	0.149
CEA(< 5vs ≥ 5 ng/mL)	1.289	0.527–3.154	0.578	1.042	0.467–2.328	0.919
CXCR2(low vs high)	0.209	0.061–0.715	0.013	0.259	0.088–0.760	**0.014**
**Multivariate analysis**						
Dukes stage						
IV	1.000	..	**0.004**	1.000	..	**0.001**
I vs IV	0.117	0.022–0.617	**0.011**	0.088	0.017–0.448	**0.003**
II vs IV	0.199	0.066–0.604	**0.004**	0.168	0.059–0.477	**0.001**
III vs IV	0.116	0.031–0.439	**0.002**	0.171	0.056–0.516	**0.002**
CXCR2(low vs high)	0.234	0.068–0.808	**0.022**	0.298	0.100–0.886	**0.029**

**Table 3:** Univariate and multivariate analyses of CXCR2 expression of CRC patients in validation set

Parameters	OS			DFS		
	HR	95% CI	*P*	HR	95% CI	*P*
**Univariate analysis**						
Age (< 65vs ≥ 65, year)	1.026	0.382–2.754	0.960	1.629	0.729–3.640	0.234
Gender(female vs male)	1.541	0.578–4.111	0.388	1.322	0.582–3.000	0.505
Histology						
Papillary	1.000	..	0.617	1.000	..	0.929
Tubular vs Papillary	0.539	0.120–2.411	0.418	0.842	0.196–3.623	0.817
Mucinous vs Papillary	0.898	0.126–6.395	0.915	1.076	0.150–7.708	0.942
Location						
Sigmoid+Rectum	1.000	..	0.912	1.000		0.324
Right hemicolon	0.763	0.197–2.952	0.695	0.260	0.059–1.150	0.075
Transverse colon	1.127	0.139–9.172	0.911	0.536	0.070–4.095	0.548
Left hemicolon	1.286	0.408–4.054	0.668	0.965	0.389–2.384	0.939
Dukes stage						
IV	1.000	..	**0.000**	1.000	..	**0.000**
I vs IV	0.123	0.014–1.078	**0.041**	0.106	0.012–0.897	**0.039**
II vs IV	0.017	0.002–0.147	**0.000**	0.047	0.014–0.160	**0.000**
III vs IV	0.152	0.049–0.469	**0.001**	0.105	0.036–0.306	**0.000**
Tumor size(< 5 vs ≥ 5, cm)	1.879	0.652–5.411	0.242	1.593	0.703–3.612	0.265
CEA(< 5vs ≥ 5 ng/mL)	1.224	0.456–3.288	0.688	1.201	0.544–2.653	0.650
CXCR2(low vs high)	0.162	0.021–1.229	**0.038**	0.069	0.009–0.509	**0.009**
**Multivariate analysis**						
Dukes stage						
IV	1.000	..	**0.003**	1.000	..	**0.001**
I vs IV	0.166	0.019–1.469	**0.021**	0.148	0.017–1.259	**0.040**
II vs IV	0.026	0.003–0.235	**0.001**	0.085	0.025–0.293	**0.000**
III vs IV	0.190	0.061–0.592	**0.004**	0.135	0.046–0.397	**0.000**
CXCR2(low vs high)	0.237	0.030–1.872	**0.046**	0.096	0.013–0.730	**0.024**

**Figure 2:**
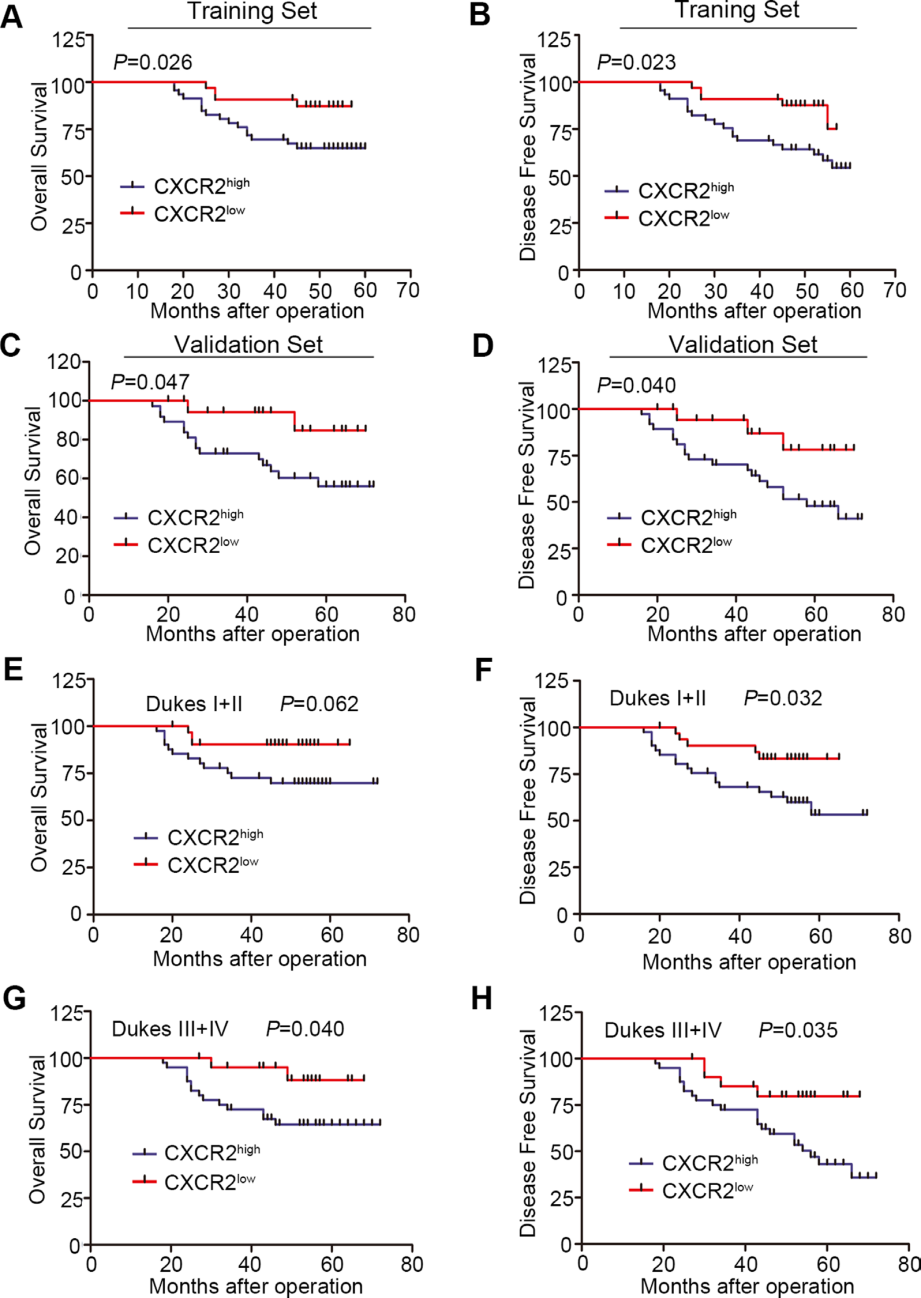
Kaplan-Meier survival analysis according to the expression of CXCR2 (**A** and **C**) Comparison of overal survival in CXCR2^high^ and CXCR2^low^ groups in both traning set (A) and validation set (C). (**B** and **D**) Comparison of disease free survival in CXCR2^high^ and CXCR2^low^ groups in both traning set (B) and validation set (D). (**E** and **F**) Kaplan-Meier subgroup analysis to evaluate prognostic value of CXCR2 by Dukes stage. OS and DFS in I + II group. (**G** and **H**) Kaplan-Meier subgroup analysis to evaluate prognostic value of CXCR2 by Dukes stage. OS and DFS in III + IV group.

Kaplan-Meier survial analysis demonstrated that patients with high expression of CXCR2 had a poor OS compared to those with low CXCR2 expression both in training set and validation set (*P* = 0.026 and *P* = 0.047 respectively) (Figure 2A and 2C). Moreover, CXCR2 was able to induce deterioration of survival status in CRC patients because CXCR2^high^ patients showed poor DFS in these two sets (*P* = 0.023 and *P* = 0.040 respectively) (Figure 2B and 2D).

To investigate whether the influence of CXCR2 expression on survival was dependent on Dukes stage, we performed a subgroup analysis by pathological stage in the overall cohort. CXCR2 expression was significantly associated with OS in Dukes III + IV CRC patients (*P* = 0.040) (Figure 2E). No significant OS difference was found between CXCR2^high^ and CXCR2^low^ patients with Dukes I + II stage (*P* = 0.062) (Figure 2F). For DFS, high-expressed CXCR2 acted as an unfavourable prognostic factor in both Dukes I + II and III + IV patients (*P* = 0.032 and *P* = 0.035 respectively) (Figure 2G and 2H). Taken together, these data above indicate that CXCR2 is an independent prognostic factor of CRC patients.

### Prognostic nomogram model of CXCR2 in CRC patients

**Figure 3:**
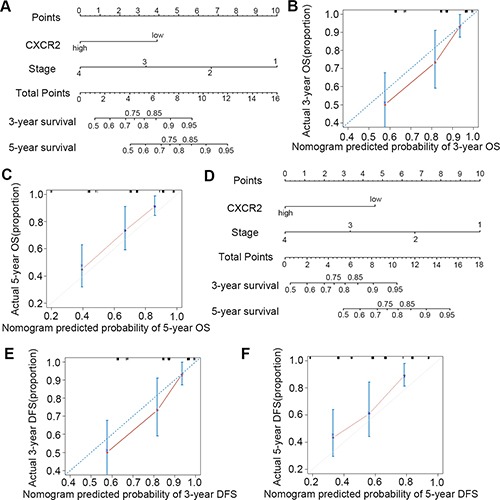
Nomogram model for the prediction of prognosis in CRC patients (**A**, **B** and **C**) prediction and calibration plots for OS; (**D**, **E** and **F**) prediction and calibration plots for DFS.

Multivariate analysis above demonstrated that CXCR2 expression and Dukes Stage were independent factors of both OS and DFS. On the basis of this, we constructed Nomogram model to predict OS and DFS at 3 and 5 years survival after surgery treatment of CRC patients by integrating all significant independent factors for OS and DFS as shown in Tables 2 and 3 (Figure 3A and 3D). The calibration plot for the predicted probability of OS and DFS at 3 or 5 years after surgery manifested an optimal consistency between the prediction by nomogram and actual survival rate (Figure 3B–3F respectively).

### Combination of CXCL5 and CXCR2 to predict prognosis on CRC patients

**Figure 4:**
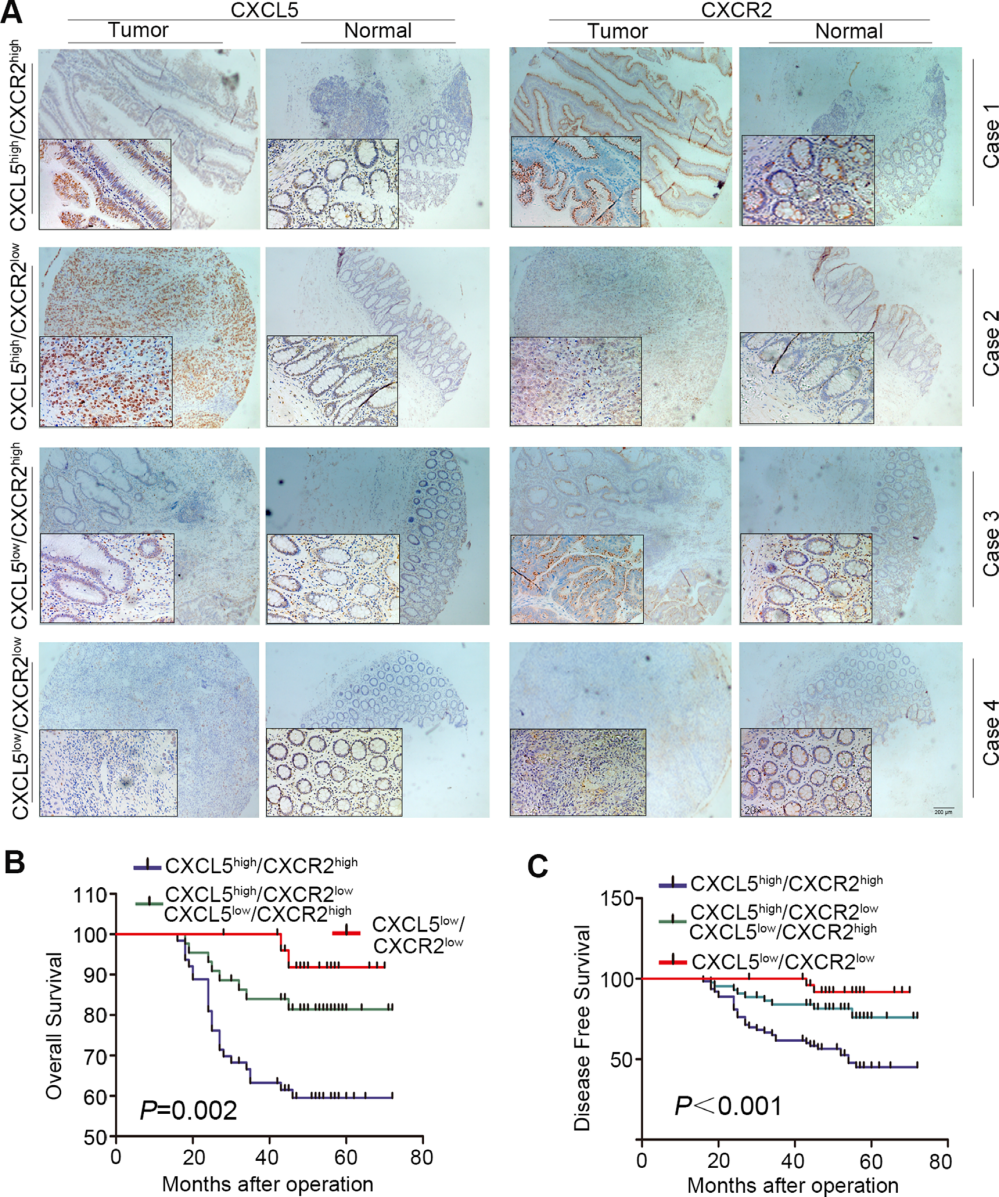
Combination of CXCL5 and CXCR2 to predict prognosis on CRC patients (**A**) expression of CXCL5 in CRC tissues; (**B** and **C**) comparison of OS (B) and DFS (C) in CXCL5^high^/CXCR2^high^ group, CXCL5^high^/CXCR2^low^ or CXCL5^low^/CXCR2^high^ group, and CXCL5^low^/CXCR2^low^ group.

CXCL5 is one of the ligands binding to CXCR2. Previously, we have revealed that CXCL5 was able to promote CRC progression at both clinical level and molecular biological level. Therefore, we combined these two factors and divided overall cohort into three groups: positive, intermediate and negative staining groups (Figure 4A). Kaplan-Meier survival analysis indicated that positive staining group has a poor prognosis compared with intermediate and negative staining groups (Figure 4B and 4C). In addition, the prognostic score composed of CXCL5 and CXCR2 had more significant value in predicting the prognosis than single CXCR2 factor both in OS and DFS (*P* = 0.002 and *P* < 0.001 respectively).

### Expression of other ligands of CXCR2 in CRC tissue

**Figure 5:**
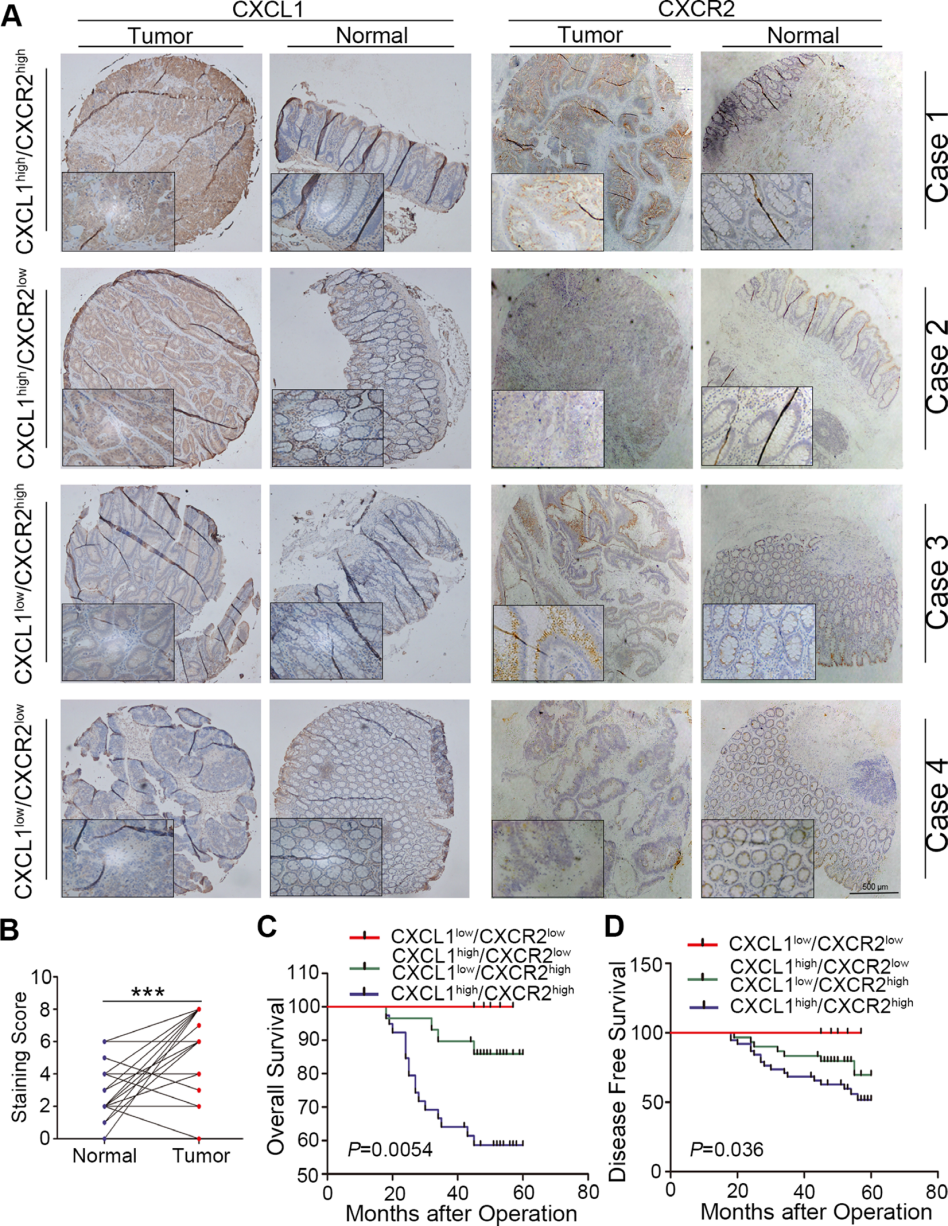
Combination of CXCL1 and CXCR2 to predict prognosis on CRC patients (**A**) expression of CXCL1 in CRC tissues; (**B** and **C**) comparison of OS (B) and DFS (C) in CXCL1^high^/CXCR2^high^ group, CXCL1^high^/CXCR2^low^ or CXCL1^low^/CXCR2^high^ group, and CXCL1^low^/CXCR2^low^ group.

CXCL1, CXCL2, CXCL3, CXCL6, CXCL7 and CXCL8 are also the ligands of CXCR2 except CXCL5. We detected the expression of these ligands using RT-PCR in 20 CRC tissue samples. RT-PCR results demonstrated CXCL1 and CXCL8 were significantly high-expressed in tumor tissues, however, there was no significant difference in the expression of CXCL2, CXCL3, CXCL6 and CXCL7 between normal and tumor tissues (Supplementary Figure 1) in RT-CPR results. And then we further verified the expression of CXCL1 and CXCL8 using immunohistochemistry. As is shown in Figure 5A and 5B, it was confirmed that CXCL1 was high-expressed in tumor tissue. Similarly, we also divided overall cohort into three groups: positive, intermediate and negative staining groups. The positive staining group has a poor prognosis compared with intermediate and negative staining groups according to Kaplan-Meier survival analysis both in OS and DFS (Figure 5C and 5D). However, not only the prognostic score composed of CXCL5 and CXCR2 had more significant value in predicting the prognosis than single CXCR2 factor in OS and DFS (*P* = 0.002 and *P* < 0.001 respectively) as we discussed above, but also the combination of CXCL5 and CXCR2 was better in the prediction than combination of CXCL1 and CXCR2 (*P* = 0.0054 and *P* = 0.036 respectively). However, as is shown in Supplementary Figures 2 and 3, IHC results showed that there was no difference in the expression of CXCL8 in CRC tissues.

## DISCUSSION

Colorectal cancer is the leading cause of death from gastrointestinal malignancy whose progression involves in various genetic mutation and modification of tumor microenvironment. Chemokines act as a messenger which links tumor microenvironment and tumor itself [12]. On the basis of relative position of cysteine residues, chemokines are subdivided in four families: CXC, CC, C and CX3C chemokines [13]. CXC chemokines can be further subdivided into ELR (Glu, Leu, Arg)+ CXC and ELR- CXC [14]. ELR+ CXC are the main ligands of CXCR2 including IL-8, CXCL1, CXCL3 and CXCL5 [15]. CXCR2 is a member of the G-protein-coupled receptor superfamily and a number of studies have demonstrated that CXCR2 plays a pivotal role in tumor angiogenesis, proliferation and invasion [16, 17]. Here in our study, we firstly report that CXCR2 is an independent prognostic risk factor in CRC patients.

As is shown above, we firstly verified the high expression of CXCR2 in CRC tissues compared with peritumoral normal tissues which is consistent with the report in other kinds of cancer [17, 18]. We also found that high expression of CXCR2 was associated with local invasion (T) and distant metastasis (M). Moreover, relative study has found that the inhibition of CXCR2 (using inhibitor SCH-527123 and SCH-479833) protects against human colon cancer liver metastasis [19]. This indicates that CXCR2 maybe able to promote CRC local and distant metastasis. Colin W. Steele *et al*. reported that CXCR2 staining was within the tumor stroma instead of the tumor in pancreatic ductal adenocarcinoma [18]. However, according to the results from IHC in our study, CXCR2 was expressed mainly along cell membrane in CRC. This was also verified by immunofluroscence staining via counterstaning stroma fibroblasts. This indicates that the function of CXCR2 in these two kinds of tumor is regulated by different pathways and the source of ligands maybe from the tumor cells.

COX regression analysis demonstrated that CXCR2 was an independent prognostic factor in CRC patients. Additionally, patients with high expression of CXCR2 tended to have a poor prognosis both in OS and DFS. Moreover, CXCR2 was able to predict 3 and 5 years survival in a Nomogram model. These data indicate that CXCR2 is an unfavorable prognostic factor with disease progression. Additionally, we found that high expression of CXCR2 predicts poor prognosis in both OS and DFS even in low-risk group (I + II). This indicates that CXCR2 can improve CRC patients’ risk stratification. We are supposed to pay attention to the low-risk CRC patients(early Dukes stage) with high expression of CXCR2. Because high expression of CXCR2 tends to predict poor prognosis in these patients.

CXCL5 is a ELR+ CXC chemokine which promotes angiogenesis through interaction with its specific receptor CXCR2 [20]. In addition, in our previous study, we have demonstrated that high expression of CXCL5 was capable of promoting CRC progression. Therefore, we combined CXCL5 and CXCR2 to analyse their correlation with patinets’ prognosis. Results indicated that CXCL5^high^/CXCR2^high^ group has worse OS and DFS compared with CXCL5^high^/CXCR2^low^ or CXCL5^low^/CXCR2^high^), and CXCL5^low^/CXCR2^low^ groups. This indicates that combination of CXCL5 and CXCR2 may have better predictive ability to unfavorable prognosis.

There are many other ligands of CXCR2 such as CXCL1, CXCL2, CXCL3, CXCL6, CXCL7 and CXCL8, we also detected their expression in CRC tissues. Our results showed that only CXCL1 was high-expressed in CRC tissues. CXCL1, also called GROα, is a secreted interleukin-like molecule that interacts with the CXCR2 G-protein-coupled receptor. Yu Wen *et al*. [21] has revealed the expression of CXCL1 in CRC tissues and discussed its function on CRC cell progression. They found that CXCL1 was higher in primary adenocarcinomas (*n* = 132), adenomas (*n* = 32), and metastases (*n* = 52) than in normal colon epithelium(*P* < 0.001). These results were also confirmed by us. In addition, we also found that CXCL1^high^/CXCR2^high^ group has a poor prognosis compared with intermediate (CXCL1^high^/CXCR2^low^ or CXCL1^low^/CXCR2^high^) and negative staining groups (CXCL1^low^/CXCR2^low^) according to Kaplan-Meier survival analysis both in OS and DFS. Combination of CXCL1 and CXCR2 also had more significant value in predicting the prognosis than single CXCR2 factor in OS and DFS. However, CXCL5 and CXCR2 was better in the prediction than combination of CXCL1 and CXCR2.

Taken together, our present study indicated that CXCR2 expression is a promoter of CRC local as well as distant metastasis and unfavorably associated with CRC patients’ prognosis. Moreover, CXCR2 can stratify high-risk patients especially in normally early stage low-risk CRC patients. Therefore, CXCR2 maybe used as a novel prognostic factor in patients. The major limitations of our study are the relatively small size of CRC patients cohort. A multicenter, prospective study is needed to validate these results in a larger population in the future.

## MATERIALS AND METHODS

### Patients and follow-up

Ethics approval for use of human specimen in our study was obtained from the Biomedical Ethics Committee of Ruijin Hospital, Shanghai Jiaotong University School of Medicine. The cohort of 134 colorectal cancer patients is enrolled in our study. All these patients, without other malignant tumors, were diagnosed specifically by pathology as CRC (before or after surgery) and treated with laparoscopic surgery in Minimally Invasive Surgery Centre, Ruijin Hospital. All the tumor tissues and paired peritumoral normal tissues contained in this cohort were collected from patients undergoing operation from 2010 to 2011. Clinical and pathological data including age, gender, histology, tumor location, tumor size, TNM stage and CEA level were collected. All the patients who had received preoperative treatment such as radiation or chemotherapy were excluded. Pathological staging of CRC tumor was performed in accordance to the TNM classification [5]. The follow-up data were acquired at 2-month intervals through outpatient visits, telephone calls, or office visits. The follow-up data were ceased in August 2015.

### Tissue microarray and immunohistochemical staining

Fresh specimens collected were immediately fixed by 4% formaldehyde after dissection and embedded with paraffin. Tissue microarray were manufactured by Shanghai Outdo Biotechnology Corporation. The staining of tissue microarray was conducted according to the manufacturer's protocol. Briefly, after being dewaxed and hydrated, the microarray were antigen-retrieved by microwaving in citrate buffer (10 mM citric acid, pH 6.0), blocked in 5% animal serum, and incubated with anti-CXCR2 primary antibody(dilution, 1: 250) overnight at 4°C. Next detection was stained for 10 min at 37°C by using secondary biotinylated anti-rabbit antibodies at a 1:100 dilution followed by streptavidin-HRP. Specimans were developed by DAB and the nuclei was counterstained by Hematoxylin. The sections were photographed under a microscope.

### Immunohistochemical score

**Figure 6:**
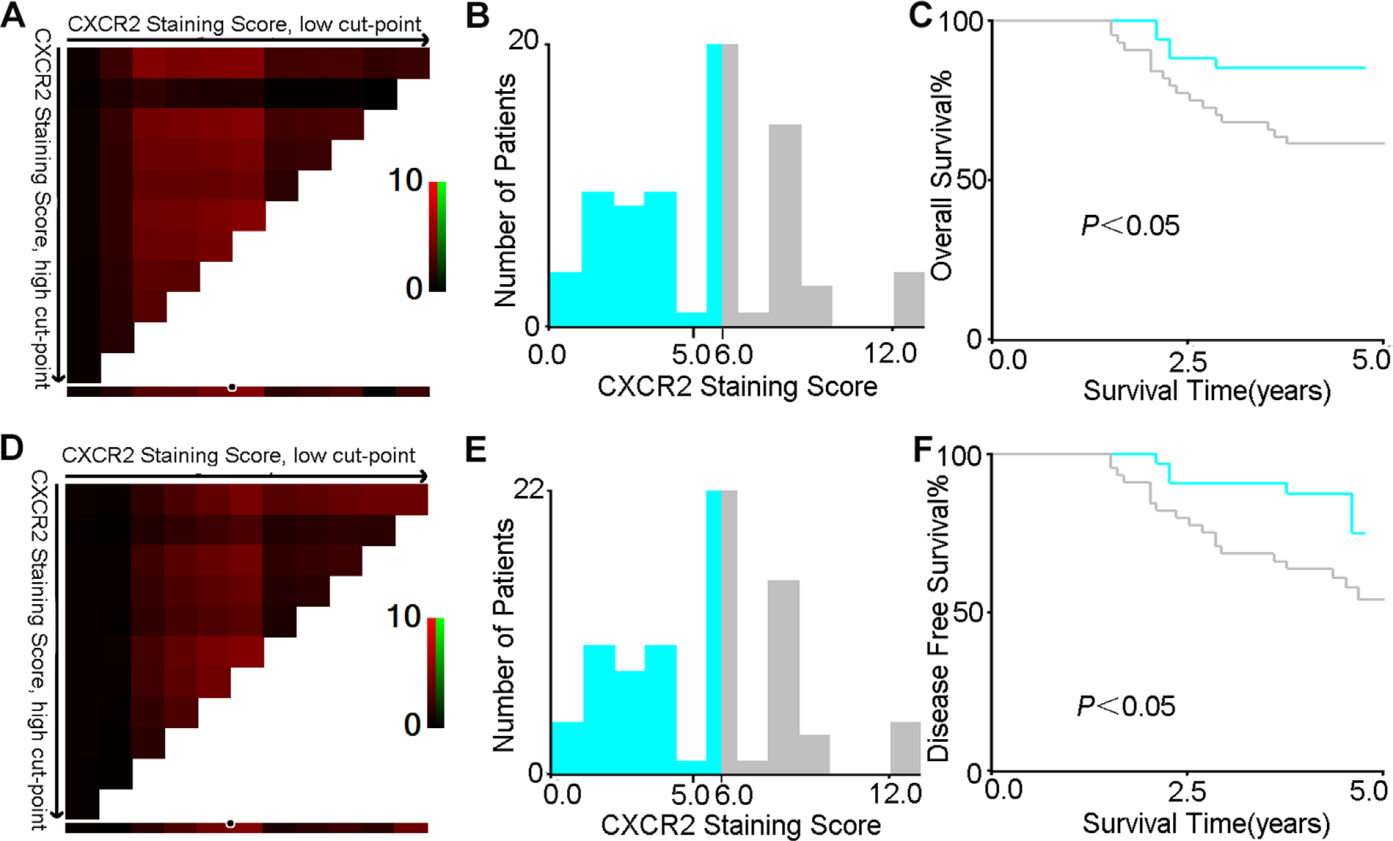
X-tile analysis of survival data in CRC patients reveals a continuous distribution based on CXCR2 staining score The plot shows the χ2 log-rank values produced when dividing the cohort with one cut-point, producing high, and low subsets. The X-axis represents all potential cut-points from low to high (left to right) that defines a low subset, whereas the Y-axis represents cut-points from high to low (top to bottom), that defines a high subset. Red coloration of cut-point indicates an inverse correlation with survival, whereas green coloration represents direct associations (**A** and **D**, for OS and DFS respectively). The optimal cut-point occurs at the brightest pixel (red). The cut-point highlighted by the white circle in A and D is shown on a histogram of the entire cohort (**B** and **E**, for OS and DFS respectively), and a Kaplan-Meier plot (**C** and **F**, for OS and DFS respectively, low subset grey, high subset light green).

Intensity of immunohistochemical staining of CXCR2 in tumour tissue was scored by two independent pathologists according to semi-quantitative immunoreactivity scoring (IRS) system [22]. Intensity of immunostaining was scored as 0 (no immunostaining), 1 (weak immunostaining), 2 (moderate immunostaining) and 3 (strong immunostaining). The percentage of immunoreactive cells scoring was documented as 0 (none), 1 (< 10%), 2 (10–50%), 3 (51–80%) and 4 (> 80%). The intensity of immunostaining score and the percentage of immunoreactive cells score were multiplied to generate IRS ranging from 0 to 12 for each tumour. The optimum cut-off value 6 was calculated by using score X-tile software version 3.6.1 (Yale University School of Medicine, USA) based on the association with the patients’ overall survival. IRS more than or equal to 6 was regarded as high expression and less than 6 was regarded as low expression of CXCR2 (Figure 6). For survival analysis based on the combination of CXCL5 and CXCR2, cut-off value of CXCL5 staining was set to 4.5 using X-tile software. On the basis of this, tumors with both positive staining of CXCL5 and CXCR2 were regarded as positive staining (CXCL5^high^/CXCR2^high^), those with positive staining of CXCL5/negative staining of CXCR2 or negative staining of CXCL5/postive staining of CXCR2 were regarded as intermediate staining (CXCL5^high^/CXCR2^low^ or CXCL5^low^/CXCR2^high^), and those with both negative staining of CXCL5 and CXCR2 were regarded as negative staining (CXCL5^low^/CXCR2^low^). For survival analysis based on the combination of CXCL1 and CXCR2, the cut-off value setting and IHC grouping were performed at the same way.

### Immunofluorescence assays

For the immunofluorescence assays, cryosections of CRC were fixed with 4% paraformaldehyde for 10 minutes. After rinsing with PBS, the cells were permeabilized with 0.1% Triton X-100 for 10 minutes at room temperature. The cryosections were then washed three times with PBS and blocked with 5% BSA for 1 hour at room temperature. The sections were incubated with the primary antibody at 4°C overnight(CXCR2, Rabbit, 1:100, Abcam, UK; αSMA, 1:100, Abcam, UK). After that, each section was washed 3 times with PBS and incubated with the iFluorTM 594 goat anti-mouse or rabbit antibody (AAT Bioquest, USA) for 2 hours at 37°C. After washing with PBS, diamidino phenylindole (DAPI, Santa Cruz, USA) was used to counterstain the nucleus. The results were visualized using a laser scanning confocal microscope (Zeiss, Germany).

### Statistical analysis

All the statistical analysis was performed by SAS 8.0 or SPSS 16.0 or R software 3.1.2(R Core Team). The significance of the relationship between CXCR2 expression level and clinical features was tested by Pearson χ^2^ test and Fisher exact probability method. Overall Survival and Disease Free Survival curves were ploted using Kaplan-Meier method and differences between two groups were determined using log-rank test. Univariate and multivariate analyses were performed using the Cox model of proportional hazards. A Nomogram model was formulated based on the results of multivariate analysis and plotted using R software. *P* < 0.05 was considered to be statistically significant.

## SUPPLEMENTARY MATERIALS FIGURES AND TABLES


