# Supplementary material for: Overexpression of CXCR2 predicts poor prognosis in patients with colorectal cancer
Source: Oncotarget. 2017 Mar 10;8(17):28442–54. doi: 10.18632/oncotarget.16086 (PMC5438662; doi:10.18632/oncotarget.16086)
Supplement: Supplementary file 1 [file oncotarget-08-28442-s001.pdf]

Overexpression of CXCR2 predicts poor prognosis in patients with colorectal cancer

Supplementary Materials

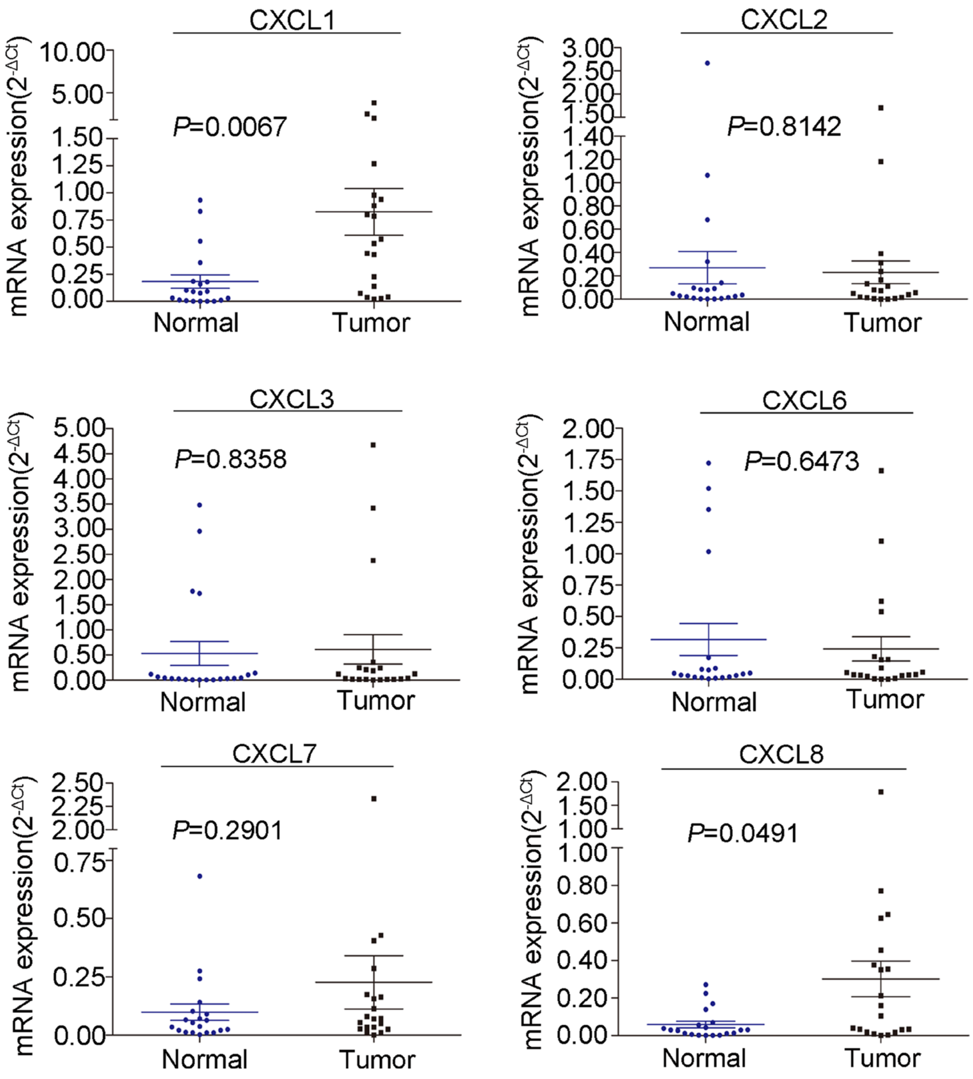

Supplementary Figure 1: RT-PCR results of CXCL1, CXCL2, CXCL3, CXCL6, CXCL7 and CXCL8 in 20 CRC tissue samples. RT-PCR results demonstrated CXCL1 and CXCL8 were significantly high-expressed in tumor tissues, however, there was no significant difference in the expression of CXCL2, CXCL3, CXCL6 and CXCL7 between normal and tumor tissues.

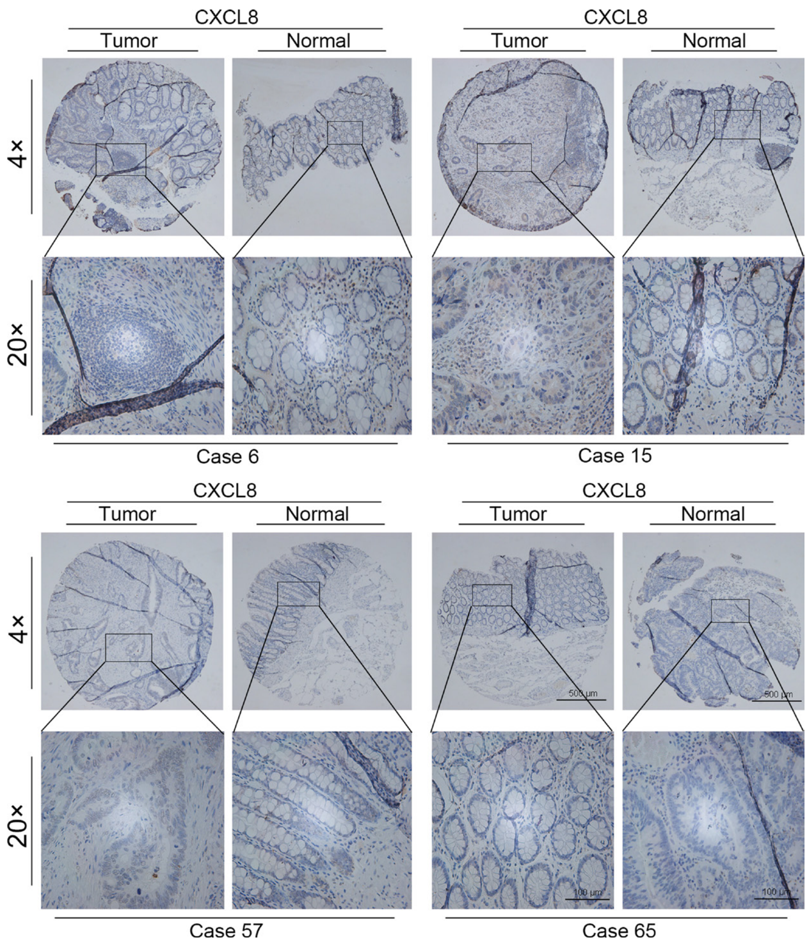

Supplementary Figure 2: The expression of CXCL8 in CRC tissues using immunohistochemistry. There is no difference in the CXCL8 expression.

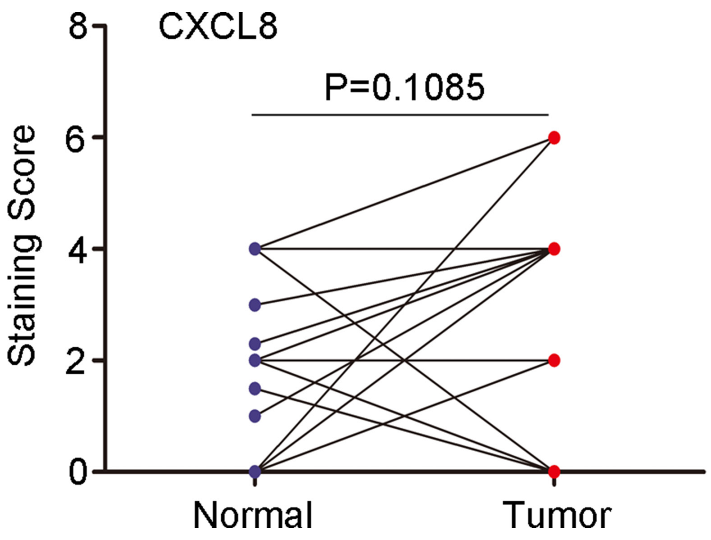

Supplementary Figure 3: Statistical analysis demonstrates that there is no difference in the CXCL8 expression.
